# Modeling Predictors of Medication Waste Reduction Intention in Algeria: Extending the Theory of Planned Behavior

**DOI:** 10.3390/ijerph20166584

**Published:** 2023-08-16

**Authors:** Kamel Mouloudj, Anuli Njoku, Dachel Martínez Asanza, Ahmed Chemseddine Bouarar, Marian A. Evans, Smail Mouloudj, Achouak Bouarar

**Affiliations:** 1 Department of Commercial Sciences, College of Economic, University Yahia Fares of Medea, Medea 26000, Algeria; kmouloudj@yahoo.fr (K.M.); shemseddine26000@gmail.com (A.C.B.); smail.mouloudj@yahoo.com (S.M.); 2 Department of Public Health, College of Health and Human Services, Southern Connecticut State University, New Haven, CT 06515, USA; evansm7@southernct.edu; 3 Department of Scientific-Technical Results Management, National School of Public Health (ENSAP), Havana Medical Sciences University, Havana 10800, Cuba; dachelmtnez@infomed.sld.cu; 4 Department of Medicine, College of Medical, University Saad Dahleb Blida 1, Blida 09000, Algeria; achouakbr122@gmail.com

**Keywords:** medication ecotoxicity, environmental health, moral obligation, pharmaceutical waste, perceived risk, unused medications, medication waste, environmental pollution, Africa, theory of planned behavior

## Abstract

COVID-19 caused an increase in the demand for medications, which led to an increase in pharmaceutical waste and there is no doubt that this contributes to environmental pollution. Hence, it became necessary to search for how to protect and improve the environment by encouraging the behavior of medication waste reduction. Accordingly, this study aims to investigate the factors affecting intentions to reduce medication waste. Considering this, we develop an extended theory of planned behavior (TPB) framework by incorporating the constructs of moral obligation, environmental awareness, and medication waste risk perception. Using the convenience sampling method and based on a self-administered questionnaire, a total of 225 usable responses were collected in five Algerian cities. The results showed that positive attitudes (β = 0.316, *p* < 0.001), moral obligation (β = 0.291, *p* < 0.001), environmental awareness (β = 0.227, *p* < 0.001), perceived behavior control (PBC) (β = 0.151, *p* = 0.001), greater perceived risks (β = 0.127, *p* < 0.001), and subjective norm (β = 0.096, *p* < 0.05) significantly and positively influence the medication waste minimization intention. Furthermore, our analyses revealed that the extended TPB model explained 73.40% of the intention variance. In conclusion, we have explored the intentions, and there may be a gap between intent and actual behavior. Therefore, we recommend future studies to examine the factors affecting the actual behavior of medication waste reduction and to investigate environmental ethics and religious commitment as predictors of waste reduction intentions.

## 1. Introduction

In recent years, the volume of consumption, production [1], and waste of medications products [2] has witnessed remarkable growth. Furthermore, with the emergence of the COVID-19 pandemic, the demand for medications grew [3,4], the amount of medication stored in homes has increased [5], and the volume of medications consumption also surged [3,6], which has caused a growth in the volume of medication waste [4,6]. Unfortunately, the most worrying thing is that all stages of the medication life cycle (production, consumption, and disposal) contribute to environmental contamination [7]. Medication waste is defined by Mohammed et al. [8] (p. 1) as “drugs and medicines that can no longer be used”. Previous research mentioned that the improvement of the patient’s condition or recovery (sometimes due to over-prescribing), forgetfulness, the emergence of side effects, changing the dose or medication, patient death, as well as the medication expiration are the most important reasons leading to medication wastage (unused or expired) in homes [9,10,11,12]. In addition, delivering and selling near-expired medications can cause them to be wasted [2,13].

The literature on waste has revealed that many patients do not use all of the medication they buy [14]. As a result, a large part of the medications ends up in the trash, and many are completely unused and not expired [11]. Practically, many households keep significant amounts of (expired, unused, and/or unwanted) medications in their homes [1,15,16,17], and sometimes the storage method is wrong; when they want to get rid of unnecessary medications, households often resort to throwing them in the garbage, toilets, sinks, or sewage channels [15,17,18], and a small percentage of them are returned to unused/expired medication take-back points or pharmacies (for example, approximately 11% and 14% in India and Saudi Arabia, respectively) [16,19].

In fact, medication waste is a complex issue, with economic, environmental, social, and ethical dimensions [15,18], and accordingly, most of the methods of medications disposal (mentioned above) fall within the irresponsible and unethical behaviors that have a serious impact on human health and the environment, including animals, water, and plants [20]. The practice of wasting medications as immoral [15] and irresponsible behavior includes the excessive purchase of medications (such as painkillers, ointments, etc.); storage of unused medications; throwing medications in rubbish, latrines, and drains; as well as donating stored medications to relatives, friends, and neighbors [10]. Moreover, these behaviors are often associated with a lack of environmental awareness and knowledge about the negative consequences and effects of wasted medications [1,18] and lower levels of perceived risk associated with wasted medications [21]. In this context, Kahssay et al. [5] found that 84.1% of the respondents in Ethiopia “had never heard/learned” about methods of safe disposal of medications. Recently, Håkonsen et al. [22] stated that despite the improvement in awareness of the serious harms of medications, studies on the influence of this awareness in consumer behavior are very few. Savari and Gharechaee [23] found that the risks perception posed by “chemical fertilizers” influences the intention to use them safely. Cortés et al. [24] (p. 1) stated that “a population’s perception of environmental health hazards is a powerful driving force for action and engagement in safety and health behaviors and can also inform the development of effective and more sustainable environmental health policies”. Luís et al. [21] argue that the environmental risk perception of medications can contribute to solving the waste problem.

Thus, the waste of medications is considered a serious problem which has serious negative consequences on the environment and society [13,15,20,25,26,27] and public health [6,28,29], and leads to economic and financial losses [26,30] that greatly affect the health care budget [19,31] in various countries of the world. Accordingly, solving this problem requires the concerted efforts of many stakeholders [31], and taking appropriate measures to reduce medication waste [4]. According to Smale et al. [31], various stakeholders can contribute effectively to preventing medication waste. For example, with regard to consumers, they state that “patients’ awareness of medication waste must be increased to stimulate conscious medication-ordering and to create willingness for participation in waste-minimizing interventions” (p. 1). In line with this, and in order to mitigate the waste severity, many countries have established medication waste take-back systems [6,18,27] so that consumers of medications can cooperate with pharmacists to play important roles. However, some of these systems failed to achieve the desired goals [12] for several reasons, including a lack of awareness among society’s members [15,18,32]. On this basis, research in this field has attracted the attention of researchers, decision-makers, and international organizations in both developed and developing countries [6].

Despite it all, current research has focused on investigating issues related to the medication waste reality, such as the examination of medication waste volume [2], medication waste disposal [6,9,12,16,17,33,34], medication waste practices [13,27], knowledge, awareness, and attitudes towards wasting medications [1,28,35], causative factors of medication waste [14], and determinants of medications storage [5,36,37]. Moreover, other studies have focused on medication waste management [4,8,25,26]. However, little attention has been paid to the medication waste reduction behavior (e.g., refs. [30,31]), especially from the consumers’ perspective (e.g., refs. [32,34,38,39]). Accordingly, there is a research gap in understanding what drives medication consumers to reduce medication waste.

Despite Algeria’s efforts in the field of medication waste management, it still needs to reassess the situation and involve all parties, especially medication consumers and pharmacists, in a specific strategy to reduce the various types of pharmaceutical waste. The few studies that dealt with the issue of medication waste management in Algeria pointed to some facts, including an increase in medication waste, lack of training in medication disposal, and the absence of a clear medication waste management strategy [25,40]. In terms of health policy, the Algerian government provides free medication to insured patients with chronic diseases (such as diabetics) and this may increase the level of wastage of medications for these diseases in light of the lack of health and environmental awareness. On the other hand, in Algeria, as in other African and Arab countries, there is no system for recovering wasted medications, and what mainly exists is some voluntary behavior to return medications to pharmacies or charities. Chisholm et al. [25] (p. 1149) have stated that “some countries, including Ethiopia, Botswana, Nigeria, and Algeria, do not have national guidelines in place to adhere to the correct disposal of such wastage”. Furthermore, there is a scarcity of research conducted in Africa in general, and Algeria in particular, that focuses on medication waste reduction. To the best of our knowledge, this is the first research in Algeria and one of the few in Africa that seeks to examine the intentions to reduce the waste of medication from the perspective of individuals (patients and their families).

Given the above arguments, medication waste behavior can be controlled (at the household consumer level) by investigating antecedents of medication waste reduction intentions and behavior, and this allows for minimization of the negative effects of medication waste on the environment and public health. As such, this research aims to model consumers’ intentions to reduce medication waste by extending the lenses of “the planned behavior theory” (TPB) model developed by Ajzen [41] with three additional constructs (i.e., moral obligation, environmental awareness, and risk perception of medication waste) in the context of Algeria as a developing country. Accordingly, our focus in this study was to understand how consumers (patients) are incentivized to reduce their medication waste.

## 2. Theoretical Background and Hypotheses Development

### 2.1. Extended TPB in the Waste Reduction Context

According to the TPB lens developed by Ajzen [41], perceived behavior control (PBC), along with attitudes and subjective norms, can be employed to predict the individuals’ behavioral intention directly. Intention was defined by Mouloudj and Bouarar [42] (p. 208) as “the extent of the individual’s willingness to complete the behavior”. In this context, Alhamad and Donyai [43] (p. 1) concluded that “TPB has been widely used inside and outside of health-related research and it was found to have more accurately defined constructs, making it helpful in studying medicines reuse behavior”. Scientific evidence shows that the TPB model was successful in predicting intentions in several settings, but that evidence also demonstrated that incorporating additional constructs can be more useful in improving the prediction level (e.g., refs. [42,44,45,46]). In the same context, most of the studies that investigated intentions and behavior to reduce waste used an extended version of the TPB model instead of the original version. For instance, TPB has been extended to investigate the intentions to reduce food waste [47,48,49,50], plastic waste [51], waste-sorting [45,52,53,54], and energy consumption [55]. In addition, Aruta [56] used the extended TPB to explore intentions to reduce plastic use as one of the plastic waste reduction practices. However, in the area of behaviors associated with reducing medication waste, very few studies have employed the TPB or extended TPB to investigate intentions to reduce medication waste (e.g., refs. [34,38,39,43]). Regardless, according to the results of most of these studies, TPB better predicts waste reduction intentions or behavior.

In our study, waste reduction behavior is responsible environmental behavior refers to various actions undertaken by consumers (patients) to avoid or minimize improper disposal practices of unused and/or expired medications. These actions include stopping the discard of unwanted medication in the trash, drain, or toilets, and alternatively, it can be donated to charities, placed in medication dropboxes, or returned to pharmacies, physicians, hospitals, or medication disposal sites (if available) (See for example, ref. [31]). Therefore, in order to predict waste reduction intentions, we extended the TPB with three other constructs, specifically, moral obligation, environmental awareness, and risk perception. Logical arguments for incorporating these three constructs into the TPB model include: (1) undoubtedly, waste reduction behavior is ethical behavior, and therefore may be affected at the moral obligation level. Ajzen [41] explained that integrating the “perceived moral obligation” with TPB constructs, especially in the context of ethical behaviors, can improve the TPB model’ predictive power; (2) environmental awareness is the second variable that we have incorporated into TPB. Waste reduction behavior can be perceived as an environmentally friendly behavior, and therefore may be influenced by environmental awareness and knowledge. Lv et al. [32] argue that people with strong environmental awareness are more willing to engage in proper medication return behavior. Therefore, we expect that with a higher level of environmental awareness, people will make more efforts to minimize medication waste; (3) waste reduction behavior is both healthy and preventive behavior, and as such can be strongly influenced by perceived risks. When people perceive risks, they often avoid the behavior leading to those risks. For example, the perceived risk of contracting COVID-19 reduced the behaviors of international travel [57] and negatively affected volunteer intention as well [42]. Some studies have supported a significant link between risk perception and behaviors associated with reducing medication waste [26,58]. Thus, increasing the level of perceived medication waste risk is expected to avoid or minimize its waste.

### 2.2. Hypotheses Development

#### 2.2.1. Attitudes toward Reducing Medication Waste

According to Ajzen [41] (p. 188), attitude refers to “the degree to which a person has a favorable or unfavorable evaluation or appraisal of the behavior in question”. In our study, attitude refers to the extent to which a consumer has favorable (like) or unfavorable (dislike) medication waste reduction behaviors. In the waste literature, attitudes have been proven to be a strong antecedent of intention to waste minimization among tourists [59,60]. Likewise, positive attitudes towards waste reduction have been demonstrated to be an antecedent of intention to reduce plastic waste [51], dispose of unused medications [39], and food waste reduction (e.g., refs. [48,49,61,62]). Tang et al. [63] found that environmental attitudes positively affect waste-classification behavior. Moreover, Aruta [56] (2022) revealed that positive attitudes significantly predicted intentions to avoid plastic waste by reducing the use of plastic. Hajj et al. [28] found that positive attitudes toward correct (unused or expired) medication disposal were significantly affected by higher knowledge. A study conducted by Alhamad and Donyai [38] revealed that attitudes positively affect the intention to reuse medications (i.e., returned unused medications to pharmacies) as one of the solutions to reduce medication waste. However, conversely, Akhound et al. [55] revealed that attitudes do not significantly predict employees’ intention to reduce energy consumption. Also, Xu et al. [54] found that attitude has an insignificant effect on waste separation intention. Based on TPB’s proposition and previous empirical evidence, we developed the first hypothesis.

**H1.**  *Attitudes have a positive effect on the intention to reduce medication waste*.

#### 2.2.2. Subjective Norms

Subjective norm is the second construct in TPB, which can be defined as “the perceived social pressure to perform or not to perform the behavior” [41] (p. 188). In our study, a subjective norm refers to the level of social pressure exerted by individuals (such as doctors, pharmacists, celebrities, etc.) and groups (such as environmental protection associations and religious institutions) to minimize medication waste. In the waste literature, several studies have shown that waste minimization intentions were influenced by subjective norms [48,60,62]. Alhamad and Donyai [38] found that subjective norms are the strongest antecedent to intentions to re-use medication compared to attitudes and PBC. Refs. [54,64] found that subjective norm has a significant effect on waste separation intention. In Taiwan, Fang et al. [53] also found that social norms have a significant effect on waste reduction among visitors. Nevertheless, a study by So et al. [51] showed that subjective norms had a non-significant influence in Hong Kong on plastic waste reduction intentions. Ref. [49] also proved that subjective norms did not play a significant role in predicting intentions to minimize food waste. In light of the TPB’s proposition and discussion above, we developed the second hypothesis.

**H2.**  *Subjective norms have a positive effect on the intention to reduce medication waste*.

#### 2.2.3. Perceived Behavior Control (PBC)

The next key determinant of the TPB is PBC, which define as “the person’s perception of the ease or difficulty of performing the behavior of interest” [41] (p. 183). Hence, in our research, the PBC refers to the individual’s confidence in his/her ability to reduce medication waste. Thus, inadequate facilities to return unwanted medications and the high cost (both financial and psychological) can be barriers to waste reduction behavior. Easy access to “unused medication collection programs” plays an important role in engaging in waste reduction behavior [7]. In this context, Chong et al. [26] mentioned that the absence or lack of a medication “return service” can lead to medication waste behavior. In addition to the above, many studies previously indicated a positive relationship between PBC and waste minimization intentions [38,48,49,51,56,60,61,62]. In India, Matharu et al. [65] reported that PBC is the strongest predictor of food waste reduction behavior. However, Akhound et al. [55] emphasized that PBC has an insignificant effect on the intention to reduce energy. As ref. [54] also revealed that PBC is not a significant predictor of waste separation intention. In light of the TPB’s proposition and the literature reviewed, we developed the third hypothesis.

**H3.**  *PBC has a positive effect on the intention to reduce medication waste*.

#### 2.2.4. Perceived Moral Obligation

Personal moral obligation refers to “an individual conducting a particular behavior based on his moral responsibility or obligation” [66] (p. 108). In fact, wasting medications reflects wasting money (i.e., money loss), and this contradicts the moral principles of many people. In this context, the study of ref. [49] indicated that people who perceived food waste as money waste felt that they were morally obligated to reduce their waste. Saphores et al. [67] found out that moral norms can produce an important effect on recycling electronic waste (e-waste) behavior. In a paper by Wang et al. [60], the personal norm is proved to be the most important contributor to tourists’ waste reduction intentions. Similarly, Ref. [62] found that moral norm affects the intentions to avoid food waste. In addition, previous studies have confirmed that moral obligation is an important antecedent for predicting intention to reduce food waste [68], sort waste [69], use “chemical fertilizers” safely [23], reduce energy in workplaces [55,66], recycle [70], purchase energy-saving appliances [71], return medications appropriately [32], as well as engage in volunteering activities [42]. Moreover, Xu et al. [54] found that perceived moral obligation has a significant effect on the attitude toward waste separation. Therefore, we propose the fourth hypothesis.

**H4.**  *Personal moral obligation has a positive effect on the intention to reduce medication waste*.

#### 2.2.5. Environmental Awareness of Negative Consequences

Environmental awareness about the negative consequences of medication waste can be related to knowing the amount of medication waste and knowing the most important negative damage that medication waste causes to water, soil, animals, and human health, in addition to knowing (even if simply) about how to avoid such damages. Therefore, in medication waste management, environmental awareness can play an important role in avoiding medication waste behavior as an environmentally responsible behavior. Fan et al. [64] found that consequence awareness has a positive impact on waste sorting intention. According to Tang et al. [63], both education and environmental knowledge positively affect waste-classification behavior. In addition, ref. [67] showed that e-waste toxicity knowledge affects the recycling e-waste behavior. In a recent study on food waste in Indonesia, Purwanto et al. [72] find out that intention to minimize waste is significantly determined by environmental awareness.

In the context of medication waste, sufficient awareness of the environmental hazards can be a driver to engage in waste reduction behavior and may increase people’s willingness to pay a premium for the proper disposal of excess medication as well. For instance, ref. [7] revealed that environmental awareness and education level influence willingness to engage in unused medication disposal programs and to pay for a medication disposal program, respectively. Interestingly, a study by Wang et al. [29] found that willingness to pay for reducing pharmaceutical waste was influenced by general environmental concerns. Ref. [4] showed that educational level has a significant effect on the willingness to return medication waste to pharmacies in South Africa. Huang et al. [36] reported a lack of awareness regarding the storage and disposal of medications in China, and they confirmed the possibility of improving awareness through education and highlighting the benefits of proper storage and disposal. Also, Attiq et al. [73] confirmed that environmental awareness and environmental knowledge may be important predictors of waste reduction intentions. In this regard, we expect that as the environmental awareness about medication waste increases, an individual’s intention to minimize waste increases. Thus, we suggest the fifth hypothesis.

**H5.**  *Environmental awareness has a positive effect on the intention to reduce medication waste*.

#### 2.2.6. Medication Waste Risk Perception

In the medication waste field, we can define risk perception as assessing the risks associated with poor disposal practices of excess medications and knowing the negative consequences of those actions on both health and the environment. The previous literature revealed that medication waste contributes to environmental damage (such as water and air poisoning) which negatively affects living organisms and human health [19,21]. In some cases, the medication waste can cause air poisoning, which results in or exacerbates various respiratory diseases (asthma, chronic obstructive pulmonary disease, emphysema). The risk of taking some medications can result in death from inappropriate administration or misuse [6,19]. Alajärvi et al. [74] found that the availability of information about the environmental risks of medications affects the willingness to pay to adopt an eco-friendly “pharmaceutical policy”. Chong et al. [26] reported that the risk of child poisoning when swallowing medications (unused or expired) is one of the reasons for returning medications to pharmacies as one of the practices to reduce medication waste. On the other hand, some individuals donate stored medications to others, and this can have serious consequences for those who consume them because those medications may be inappropriate or spoiled. In their study, Michael et al. [17] concluded that the inadequate disposal of medication leads to an increase in potential risks of environmental pollution and increases the potentiality of human and animal ingestion of toxic medication waste. Ref. [58] revealed that health risk perception directly influences proper medication disposal behavior, and indirectly is an influence via intention. However, Bound et al. [75] did not find any empirical evidence of a clear connection between perceived risks and responsible disposal behavior of unused medications. Accordingly, we hypothesize that individuals are likely to be more concerned about reducing medication waste when they perceive the various risks of waste. Therefore, we propose the sixth hypothesis.

**H6.**  *Medication waste risk perception has a positive effect on the intention to reduce medication waste*.

## 3. Materials and Methods

### 3.1. Questionnaire Design

Data were collected by using a self-administered questionnaire. The questionnaire consisted of two sections: the first section was devoted to collecting demographic data of the respondents such as gender, age, educational level, occupation, and average monthly household income. Various practices of “medication waste reduction behavior” were included at the questionnaire’s beginning to make the respondents aware of this concept. To find out if the respondents had stored medications, they were asked the following question: “Do you have unwanted (unused or expired) medications in your house?”. The second section was designed to measure the study constructs. Thus, the attitudes scale was adapted from Mouloudj et al. [46]. To measure subjective norms, we used scales from Savari and Gharechaee [23] and Shi et al. [69]. PBC was measured using a scale adapted from Shi et al. [69] and Tan et al. [71]. The perceived moral obligation scale was adapted from Savari and Gharechaee [23] and Mouloudj and Bouarar [42]. As for environmental awareness, we used a scale adapted from Mouloudj et al. [46] and Obuobi et al. [68]. The perceived risk scale is derived from Savari and Gharechaee [23] and Mouloudj and Bouarar [42]. The intention to reduce medication waste scale is adapted from Shi et al. [69]. Each construct was measured with three items, except for the perceived risk construct, which was measured with four items, thus the measurement tool consisted of 22 items. The initial version of the questionnaire was formed in English, and then it was translated by three professors into Arabic. All items were evaluated using a five-point Likert-type scale ranging from 1 “strongly disagree” to 5 “strongly agree”. After completing the questionnaire design, it was sent to four experts in waste studies in order to receive their opinions on its content, after which we reformulated some items according to the suggestions of these experts. Next, we conducted a pilot study with a sample of 22 respondents to see the clarity of the measurement items. The measurement tool is shown in Table 1.

### 3.2. Sample and Data Collection

The study population included all medications consumers, aged ≥18 years, who live in five cities in northern Algeria, namely Medea, Bouira, Blida, Algiers, and Tipaza. Due to cost and time constraints, a convenience sample of 350 respondents was selected. Two researchers distributed the questionnaire, where the respondents were interviewed at several points (such as at the entrances of commercial centers, public parks, hospitals, pharmacies, and private clinics) during the period from mid-January to the end of April 2023. Filling out the questionnaire takes about ten minutes. The respondents were informed about the scientific purposes of this study, they were left free to express their opinions, and they were also informed of the freedom to complete or not fill out the questionnaire. Accordingly, all ethical requirements (voluntary, anonymous, and confidential), including informed verbal consent, were met during the data collection process. At the end of the period, we collected 238 questionnaires (response rate 68%). After sorting the answers, we found 13 invalid questionnaires because they were incomplete, and accordingly, we conducted statistical analysis on 225 questionnaires.

### 3.3. Data Analysis

In order to analyze the data, we used SPSS version 26 software (IBM Corporation, Armonk, NY, USA). After coding the data and entering it into the SPSS program, we employed descriptive statistics to calculate the means and deviations for all constructs and the percentage of respondents’ characteristics. Cronbach’s alpha coefficient was calculated to estimate the internal consistency of each construct. Normal distribution was evaluated using the skewness and kurtosis test. The multicollinearity problem was assessed using the variance inflation factor (VIF) and tolerance test. Hierarchical regression analysis was performed for hypothesis testing and *p*-value < 0.05 were considered significant. The purpose of using hierarchical regression was to evaluate the contributions of integrating the three predictors (i.e., moral obligation, environmental awareness, and risk perception) in the original TPB model. In other words, the goal was to determine the percentage of improvement in predictive power as a result of adding the three constructs.

## 4. Results

### 4.1. Respondents’ Characteristics

The respondents’ characteristics are reported in Table 2. Regarding the respondents’ gender, 143 (63.56%) were males and the rest (36.44%) were females. Moreover, the results showed that for 90 (40.00%) respondents their ages ranged between 30–45 years, 76 (33.78%) were aged between 46–60 years, 41 (18.22%) were aged between 18–30 years, and 18 (08.00%) of them were over 60 years old. Furthermore, 86 (38.22%) respondents had a secondary school or lower level of education, 74 (32.89%) had a Bachelor’s degree, and 65 (28.89%) of them had a master’s degree or above. Regarding the number of family members, the majority of the respondents 142 (63.11%) had a family size between 3–6 members, 56 (24.89%) respondents had 6 or more family members, and 27 (12.00%) of them had families consisting of one or two persons at most. As for the monthly household income level, 84 (37.3%) respondents had an income between 30,000–50,000 Algerian dinar (AD), 58 (25.78%) between 50,001–70,000 AD, 47 (20.89%) higher than 70,000 AD, and 23 (10.22%) of them had monthly income less than 30,000 AD. We found that 85.78% of the respondents had unwanted medications in their homes.

### 4.2. Descriptive Statistics

Descriptive analysis indicated that the respondents had high perceived moral obligation (M = 4.18), strong intentions to reduce waste (M = 3.85), favorable attitudes towards waste reduction (M = 3.65), high PBC (M = 3.51), and a moderate level of perceived subjective norm (M = 3.43). Nevertheless, their environmental awareness (M = 2.65) and their judgments about the risks of medication waste (M = 2.47) were low. The results presented in Table 3 reveal that, for all the variables, Cronbach’s alpha coefficient exceeded 0.70 as recommended by Henseler et al. [76], where the alpha coefficients ranged between 0.75 and 0.93. In addition, normal distribution was tested (Table 3). According to Mouloudj and Bouarar [42] (p. 212), “the normality test is an important assumption to perform a multiple regression analysis”. The skewness and kurtosis values ranged from −1.48 to +1.42, and −0.10 to +2.86, respectively, as mentioned by Ref. [42] (p. 212) which states “the data follows a normal distribution if the values of skewness range between ±2 and values of kurtosis range between ±7”. Accordingly, these results confirm that the reliability condition is fulfilled, and that the data follow a normal distribution.

### 4.3. Research Hypotheses Testing

To test the multicollinearity, the “variance inflation factor” (VIF) values and the tolerance values were calculated in the four hierarchical regression models. Table 4 reveals that the tolerance values ranged from 0.478 to 0.911, while VIF ranged from 1.098 to 2.090; this means that the tolerance values exceeded the 0.2 thresholds and the VIF values did not exceed the maximum 5 as recommended by Ref. [77]. Accordingly, there was no problem with multicollinearity.

In order to test the hypotheses, we applied “hierarchical multiple regression” by considering four models, whereas in Model 1 we tested only the TPB constructs. In Model 2, a moral obligation was integrated into Model 1; in Model 3, environmental awareness was integrated into Model 2; and in Model 4, perceived risks were merged into Model 3. The results show attitude (β = 0.362, *p* < 0.001), subjective norm (β = 0.239, *p* < 0.001), and PBC (β = 0.209, *p* < 0.001) are effective in forming an intention to reduce medication waste. As a result, H1, H2, and H3 are accepted by Model 1. The results also show that the TPB lenses explained 63.80% of the variance in medication waste reduction intentions.

In Model 2, the results indicate that attitude (β = 0.337, *p* < 0.001), moral obligation (β = 0.261, *p* < 0.001), and subjective norm (β = 0.192, *p* < 0.001), and PBC (β = 0.167, *p* = 0.001), respectively, have a significant positive effect on the intention to reduce medication waste. Therefore, H4 is confirmed by Model 2. Table 3 reveals that incorporating the moral obligation variable within TPB improved the variance in reducing waste medications intention from 63.80% to 67.80% (significant improvement by 4%).

Regarding Model 3, the results demonstrated that attitude (β = 0.304, *p* < 0.001), moral obligation (β = 0.244, *p* < 0.001), environmental awareness (β = 0.224, *p* < 0.001), PBC (β = 0.164, *p* = 0.001), and subjective norm (β = 0.107, *p* = 0.01), respectively, all exert a significant positive effect on medication waste reduction intentions. Thus, H5 is accepted by Model 3. In this model, the R^2^ value was 0.718, which means that the five constructs explained 71.80% of the variance in intention to reduce waste medications. This indicates that the integration of both “moral obligation” and “environmental awareness” within the original TPB model improved the explained variance by 8%.

Finally, based on the results of Model 4, attitude (β = 0.316, *p* < 0.001), moral obligation (β = 0.291, *p* < 0.001), environmental awareness (β = 0.227, *p* < 0.001), PBC (β = 0.151, *p* = 0.001), perceived risks (β = 0.127, *p* < 0.001), and subjective norm (β = 0.096, *p* < 0.05), respectively, had a significant positive effect on medication waste reduction intentions. Thus, H6 is supported by Model 4. In model 4, the R^2^ value was 0.734, which means that the six constructs explained 73.40% of the variance in intention to reduce waste medications. This indicates that the integration of perceived risks within Model 3 improved the explained variance by 1.60%.

## 5. Discussion, Implications and Limitations

### 5.1. Discussion

In this study, we integrated moral obligation, environmental awareness, and perceived risk into the TPB model to study the intentions to reduce medication waste in Algeria. We demonstrated that attitudes towards waste minimization behavior have a positive effect on the intention to reduce medication waste. Hence, this result suggests that people with positive attitudes are more likely to form positive intentions to reduce their medication waste. This finding is consistent with previous research findings on the relationship of attitudes with waste reduction intentions (e.g., refs. [39,44,49,51,56,59,62]). Sometimes, some medication consumers may reduce/or stop wasting medications in order to avoid wasting money. In the context of food waste, Graham-Rowe et al. [78] indicated that financial concerns about waste may cause negative feelings, and this makes it a key driver of waste avoidance. Therefore, the creation of negative financial attitudes towards wasting medications may help to adopt waste-medication-reducing behavior for financial reasons. However, for the segment of individuals who have health insurance that enables them to cover the medications costs, they cannot be addressed with money-saving messages. The same is the case with the segment of people with chronic diseases, as a result of their medications being covered by the governments of their countries. In addition, if the cost of wasting medications is small for some individuals, they cannot be persuaded to reduce their medication waste for financial reasons. In other words, various people who believe the medication waste cost is small are unlikely to be willing to engage in waste reduction behavior for economic reasons.

Our results revealed that subjective norm exerts a significant effect on the intention to reduce medication waste; however, it turned out to be the least influential construct in forming the intention to reduce waste. This means that some “informational”, “normative”, and “comparative reference groups” can exert a positive pressure on wasteful people to reduce or stop their waste. Hence, when reference groups’ members gain more trust, they gain more persuasive power, and can exert a lot of pressure on their followers (i.e., wasters). It is widely accepted, and consistent with empirical evidence, that subjective norm was an important predictor of intention to reduce waste [43,44,48,62]. In addition, Ref. [79] showed that subjective norms positively affect solid-waste-separation intention. It should be noted that because medication waste is an invisible behavior to others, the impact of social pressure on wasteful individuals can be small in some cases. Nevertheless, this result is inconsistent with the empirical evidence in some previous studies, which revealed the insignificant links between subjective norms and intentions to reduce waste in different contexts [39,49,51]. This suggests that cultural differences may lead to a difference in the importance of subjective norms in influencing the attitudes, intentions, and behavior of other individuals, as mentioned by Tan et al. [71].

The important role played by PBC in explaining individuals’ medication waste minimization intention is consistent with extant empirical evidence on the waste reduction literature [7,38,39,60,61,65]. This means that the perceived ease or difficulty of waste-reduction procedures plays a crucial role in influencing waste-reduction intentions. Vassanadumrongdee and Kittipongvises [79] claimed that solid waste-separation intention is negatively affected by perceived inconvenience. Sonowal et al. [35] believe that the reason why consumers dispose of unused medications in household waste is due to the ease of doing so and to save time. Similarly, Ref. [26] concluded that lack of facilities and high operational costs for safe disposal are the most important reasons for not engaging in medications return behavior. Moreover, Saphores et al. [67] concluded that recycling convenience is an important determinant of the recycling behavior of e-waste. Kusturica et al. [7] (p. 46) state that “the most common reason reported for not returning medicines to pharmacies or other collection sites is lack of information and awareness on the existence of available unwanted medicine collection schemes in the community”. Accordingly, it is expected that the provision of easy and less effort solutions for the disposal of medications will increase the willingness of consumers to engage in behavior to reducing medication waste. However, this result does not support the empirical results of Ref. [34], which indicated that convenience was not an important determinant of correct medications disposal behavior in UK. One of the logical explanations for this is the different levels of facilities and convenience available for the medication’s disposal from one country to another.

The results confirmed that moral obligation was positively affecting the intention to reduce medication waste, as the second strongest antecedent of intention after attitudes. This means that considering medication waste as unethical behavior generates stronger intentions to reduce waste it. In other words, people will form strong intentions to engage in medication-waste-reduction behavior when they feel a moral responsibility towards medication waste. This result corroborates the results of several previous studies in the field of waste reduction (e.g., [55,62,68]). Similar results were reported in research by Chen [44], who demonstrated that moral obligation has a significant impact on intention towards carbon reduction. In addition, this result supports the results in the works of Foon et al. [39] and Lv et al. [32], who found that the intention to properly dispose of unused medication was positively influenced by personal norms. Moreover, when moral obligation was included in the TPB model, the predictive power of the expanded model improved by 4%. This result significantly supports the finding by Ajzen [41], which suggests that incorporating “perceived moral obligation” in the TPB model significantly improves the ability to predict intention from 3 to 6%.

There is very little research that has examined the relationship between environmental awareness and medication waste reduction intention. In our study, environmental awareness was shown to significantly affect the intention to minimize medication waste. This means that when individuals have more environmental awareness about the consequences and repercussions of medication waste, they will form positive intentions towards reducing medication waste. It is clear that most of the respondents have insufficient environmental awareness about the consequences of medication waste (M = 2.65), and this is supported in the waste literature (e.g., [32]). In this context, Ref. [33] indicates that there is a lack of knowledge regarding the ways to dispose of medications. Matharu et al. [65] pointed out that raising awareness about waste among household consumers requires the use of educational efforts. Huang et al. [36] found that increased education can lead to an improvement in the awareness level about the safe disposal of medications. Moreover, Ref. [79] confirmed that knowledge on waste problems influences solid waste-separation intention. This result is concordant with result of Attiq et al. [73] who opined that environmental awareness is an important driver of waste reduction intentions, and of Hori et al. [80] who reports that global warming awareness positively impacts energy-saving behavior as a waste reduction practice, and Ref. [32] who pointed out that consequences awareness was significantly correlated with the intention to return medications appropriately. The same results were also obtained in several behaviors associated with reducing waste [7,29,63,67,72].

Finally, as expected, the perceived risk associated with medication waste affected the willingness to reduce medication waste. This means that the perception of risks about medication waste can promote waste reduction behavior. In other words, this suggests that respondents who perceived a higher risk of medication waste may be more likely to avoid or reduce waste. Similar results were found in previous studies dealing with medication waste (e.g., [26,58]. Jafarzadeh et al. [37] reported growing concerns regarding the risks of improper storage and the disposal of medications. In this context, Sonowal et al. [35] indicated that donating stored medicines to other people may threaten their health and put them at risk. Hence, medications that are not stored properly may lose their effectiveness in treatment, and their consumption may cause negative reactions, such as poisoning and even death. However, it should be noted that sometimes a higher perceived risk may be positively associated with medication wasting behavior. For example, high perceived risk of unused medication can lead to its immediate disposal in the sink or toilet [34]. In addition, familiarity with medication can also reduce perceived risk, which may discourage waste-reduction behavior. Ref. [75] (p. 305) argued that “where the medicine is familiar, such as with painkillers and antihistamines, the perceived risk is lower”. Briefly, we conclude that individuals who are under high-risk perception influence can change their positive attitudes, intentions, and behaviors towards wasting medications, and can also contribute to increasing others’ awareness of those risks through word of mouth. For instance, when parents perceive the risks associated with their young children taking medications stored in the refrigerator, they can act wisely and avoid leaving unnecessary medications at home.

### 5.2. Managerial Implications

In accordance with the rule “prevention is better than cure” and the principle of “waste hierarchy”, i.e., 3R (“reduce, reuse, and recycle”), the major sources of medication waste should be targeted. In other words, trying to stop the generation of medication waste in the first stage, based on an understanding: when and where is medication wasted? Who are the main contributors to it? For example, the following proposals could help dry up some of the medication waste sources: (1) educate people about disease prevention, (2) require pharmaceutical companies to reconsider the package sizes of some medications, and (3) establish an effective system for monitoring stored medications to avoid expiry. In addition, we suggest the following proposals to effectively boost reducing medication waste behavior:

First, with regard to attitudes, efforts should be concerted to develop a negative attitude among different stakeholders towards medication waste behavior, by pinpointing the disadvantages and the negative aspects of waste and its cost. On the other hand, to develop a favorable attitude towards waste reduction behavior and reinforce it by convincing people of its various benefits.

Second, regarding subjective norms, providing recommendations and instructions to opinion leaders (such as doctors and pharmacists) and religious leaders will play a key role in guiding, educating, persuading, and motivating the various categories of society to engage in waste-reducing medication practices; celebrities and social media influencers can also play a significant role in this regard by sending positive messages and advertising for rational behavior concerning avoiding waste behavior.

Third, concerning PBC, removing the barriers and obstacles that prevent and reduce medication waste behavior will play an effective role in encouraging many medications consumers to engage in these behaviors. For example, education and culture can help remove the cognitive barriers associated with ignorance of specific behavior to deal with unwanted medications (used or expired), thus the existence of several convenient sites for collecting unwanted medications (such as pharmacies and associations, etc.) can help reduce the effort and cost of disposing of those medications, which encourages returning behavior.

Fourth, concerning consumer awareness of the consequences of wasting medications, it would be beneficial to improve the level of people’s awareness of the environmental and health damages of wasting medications. Hence, various stakeholders (such as the Ministry of Health, hospitals, educational institutions, charities, pharmacists, and doctors) can contribute to educating people on the best ways to properly dispose of excess medications, which results in encouraging consumers to adopt environmentally friendly behaviors and avoid continuing waste. In addition, pharmaceutical companies can provide consumers with guidance on the best medication disposal behavior by placing educational labels on medication packages or including sufficient information in the medication information leaflet. They can also sponsor and pay for the costs of medication waste reduction programs.

Fifth, with regard to ethical obligation, it would be very useful to make people feel that wasting medications opposes ethical values; this can be achieved through advertising the risks of waste on human health, the environment, and the economy as a whole. Organizing periodic donation campaigns under the motto “Give the medicines you do not need to the poor” can also develop a sense of moral duty. In addition, promotional campaigns could be designed to target the most medication-wealthy segments to make them resentful about wasting too much medication when others are suffering from a shortage.

Sixth, concerning perceived risks, it would be vital to raise the level of perceived risks of wasting medications by highlighting the risks of leaving unused or expired medications at home (for example, they may cause harm to children), and the risks of throwing them in the garbage (such as harming cleaners), and the risks of throwing them into the sewage system (water pollution), etc.; avoiding these behaviors can be achieved by sending promotional messages (carrying in their content serious alerts and warnings about these risks) aimed at raising feelings of concern and anxiety of misconduct around excess medication. Enlisting the media, social networks, educational and religious institutions, and putting posters on roads and public places, for example, can also be very effective to disseminate these warnings.

Finally, associations (or any other entities) can also organize periodic visits to homes to collect waste medications as an effective solution to the problem of medications consumers’ lack of engagement in environmentally responsible behaviors due to their lack of awareness, insouciance, or ignorance of the risks associated with medications wastage. It is also very likely that providing symbolic financial incentives will contribute to encouraging many medications consumers to avoid wasting medications, especially expensive medications and medications that have a severe impact on the environment and public health. In addition, physicians can effectively contribute to reducing medication waste by prescribing the exact amount of medications needed, reducing the errors associated with prescribing medications, and avoiding writing three month prescriptions.

### 5.3. Strengths and Limitations of Study

This is one of the few studies from Africa that seeks to explore the individuals’ intentions toward medication waste reduction by expanding the lenses of the TPB model. This paper contributes to the growing discussions on pharmaceutical waste management in the context of developing countries. Notwithstanding, like other research, this research is not without some limitations. First, our extended model explained about 73% of the intention variance, which is a high percentage, so it would be beneficial to adopt the same framework in other countries. Second, our study extended TPB with three factors, so it may be useful to integrate other factors such as environmental ethics and religious commitment; or TPB can be merged with other theories such as the “Health belief model” (HBM), “Protection motivation theory” (PMT), “Norm activation model” (NAM), and trans-theoretical model of health behavior change (T-TMHBC). Third, it would be useful for future research to focus on finding innovative technological solutions to remove any barriers that may hinder the medication waste minimization behavior. Fourth, our study did not consider the possible relationships between medication waste reduction intentions and demographic characteristics. Hence, it is important to examine this in the future to increase the ability to design strategies targeting specific demographics. Fifth, in this study our focus was on the investigation of behavioral intentions. However, Mouloudj et al. [81] (p. 119) state that “the influence of behavioral intention on the customers’ actual behavior remains questionable”. Hence, we recommend future studies to examine the factors influencing the actual behavior of medication waste reduction. Lastly, since resources waste has been proven to have a huge impact on health, the environment, and the economy as well, and it has also been proven that our planet is exposed to more pollution and poisoning due to waste and other factors, we call from this rostrum (urgently and essentially) to conduct further investigations on the waste reduction behavior of various pharmaceutical products (such as cancer medications, vaccines, vitamins, cosmetics, as well as pharmaceutical packaging materials) because this can effectively contribute to adopting effective strategies to minimize waste and thus the possibility of saving our planet.

## 6. Conclusions

Despite the benefits of medicines in improving public health, their waste does as much harm to the environment, health, and the economy, as mentioned in the waste literature. Hence, based on the fact that the behavior of medicine consumers can contribute to more medication waste, and in order to understand how to involve consumers in waste management strategies, it is interesting to investigate the factors that reduce this behavior from the consumer’s point of view. For this and based on a self-administered questionnaire and a convenience sample of 225 Algerian medicine consumers, we used hierarchical regression to model the medications’ waste reduction intentions. The results of the current study revealed that the TPB model constructs (original version) were able to explain 63.80% of the variance in medication waste reduction intention. This indicates the validity of applying the TPB model in the context of waste reduction behavior. However, when the three additional constructs (i.e., moral obligation, environmental awareness, and risk perception) were combined into TPB, the extended model explained 73.40% of the variance in intentions, and this indicates the importance of including additional constructs in improving the predictive power of the original TPB. These results suggest that raising environmental awareness, warning of the dangers of wasting medications, increasing the level of ethical commitment, and fostering positive attitudes towards avoiding waste, as well as activating the role of subjective norms (such as physicians and pharmacists), have a very effective role in the formation of intentions to reduce medication waste. Finally, it seems that the success of efforts to reduce the waste of medications requires the involvement of other stakeholders, such as pharmaceutical companies, distributors, doctors, and pharmacists, among others.

## Figures and Tables

**Table 1:** Constructs and items.

Constructs	Items	Sources
Attitudes (At)	AT1—I believe that reducing medication waste is a good ethical behavior	[46]
	AT2—I think it is wise and rational to reduce the medication waste	
	AT3—I think that reducing medication waste will be useful for the environment	
Subjective norm (SN)	SN1—Most people whose opinions I value want me to avoid wasting medication	[23,69]
	SN2—If I reduce my medication waste, the people important to me will support this behavior	
	SN3—People I respect are striving to reduce their medication waste	
Perceived behavior control (PBC)	PBC1—I am confident that I can reduce my medication waste	[69,71]
	PBC2—I have time, abilities, and opportunities to reduce my medication waste	
	PBC3—I believe I am capable of reduce my medication waste	
Moral obligation (MO)	MO1—I believe I have a moral responsibility to reduce my medication waste	[23,42]
	MO2—Reducing my medication waste is based on my own moral obligation	
	MO3—I think that continuing to generate more waste medications violates my ethical principles	
Environmental awareness (EA)	EA1—Wasting medication can contribute to environmental poisoning	[46,68]
	EA2—Wasting medication can increase water and air pollution	
	EA3—I am aware that the unsafe disposal of unused or expired medications exacerbates environmental problems	
Perceived risks (PR)	PR1—I perceive I am taking some risks when I waste the expired or unused medications	[23,42]
	PR2—I am afraid that my medication waste will cause harm to other people	
	PR3—I see that wasting medications can threaten the health of my family members	
	PR4—I am always worried about the negative consequences of medication waste on the environment and people	
Intention to reduce waste (IRW)	IRW1—I will do my best to reduce my medication waste in the future	[69]
	IRW2—I definitely want to reduce my medication waste in the future	
	IRW3—I am willing to reduce my medication waste in the future	

**Table 2:** Respondents’ profiles (*N* = 225).

Characteristics	Characteristics	*N*	Percentage
Gender	Male	143	63.56
	Female	82	36.44
Age (years)	18–30	41	18.22
	31–45	90	40.00
	46–60	76	33.78
	61 and over	18	08.00
Education level	Secondary school or lower	86	38.22
	Bachelor’s degree	74	32.89
	Master’s degree or above	65	28.89
Number of family members	1 or 2	27	12.00
	3–5	142	63.11
	6 or above	56	24.89
Monthly household income level (Algerian dinar)	<30,000 AD	23	10.22
	30,000–50,000 AD	84	37.33
	50,001–70,000 AD	58	25.78
	>70,000 AD	47	20.89
	Missing	13	05.78
Storing medications at home	Yes	193	85.78
	No	32	14.22

**Table 3:** Descriptive statistics and normal distribution analysis.

Constructs	Mean	SD	Cronbach’s Alphas	Skewness	Kurtosis
Attitudes	3.654	0.829	0.892	−0.911	0.634
Subjective norm	3.434	0.869	0.937	−0.812	0.340
PBC	3.514	0.708	0.888	−0.563	−0.084
Moral obligation	4.188	0.616	0.757	−1.483	2.862
Environmental awareness	2.656	0.772	0.926	−0.147	−0.108
Perceived risks	2.473	0.750	0.872	1.427	2.321
Intention to reduce waste	3.859	0.698	0.863	−1.029	1.555

**Table 4:** Results of hierarchical regression analysis.

Models		B	t	Sig	Tolerance	VIF	F	R^2^ (Adjusted R^2^)
Model 1	(constant)	0.984	6.462	0.000			132.709 (*p* < 0.001)	0.643 (0.638)
	At	0.362	8.111	0.000	0.577	1.732		
	SN	0.239	5.632	0.000	0.581	1.721		
	PBC	0.209	3.895	0.000	0.547	1.828		
Model 2	(constant)	0.289	1.484	0.139			118.811 (*p* < 0.001)	0.684 (0.678)
	At	0.337	7.963	0.000	0.570	1.753		
	SN	0.192	4.696	0.000	0.555	1.803		
	PBC	0.167	3.262	0.001	0.534	1.872		
	MO	0.261	5.308	0.000	0.768	1.302		
Model 3	(constant)	0.190	1.039	0.300			115.151 (*p* < 0.001)	0.724 (0.718)
	At	0.304	7.585	0.000	0.558	1.792		
	SN	0.107	2.593	0.010	0.481	2.080		
	PBC	0.164	3.430	0.001	0.534	1.872		
	MO	0.244	5.305	0.000	0.765	1.308		
	EA	0.224	5.700	0.000	0.669	1.494		
Model 4	(constant)	−0.295	−1.350	0.178			104.199 (*p* < 0.001)	0.741 (0.734)
	At	0.316	8.104	0.000	0.554	1.805		
	SN	0.096	2.404	0.017	0.478	2.090		
	PBC	0.151	3.244	0.001	0.531	1.882		
	MO	0.291	6.280	0.000	0.710	1.409		
	EA	0.227	5.959	0.000	0.669	1.495		
	PR	0.127	3.788	0.000	0.911	1.098		

Note: At = attitude; SN = subjective norm; PBC = perceived behavior control; MO = moral obligation; EA = environmental awareness; PR = perceived risks.

## Data Availability

Not applicable.
